# *SlACO1* and *SlGARP* Regulate Hormone-Mediated Metabolic Profiles in Tomato Fruit

**DOI:** 10.3390/ijms27021078

**Published:** 2026-01-21

**Authors:** Yanpei Liu, Chunlu He, Liuqing Han, Haipeng Zhang, Juan Xu

**Affiliations:** 1 College of Life Sciences, Henan Agricultural University, Zhengzhou 450046, China; liuyanpei@henau.edu.cn (Y.L.); 18339146619@163.com (L.H.); 2 National Key Laboratory for Germplasm Innovation and Utilization of Horticultural Crops, Huazhong Agricultural University, Wuhan 430070, China; m15527429097_2@163.com; 3 College of Horticulture, Henan Agricultural University, Zhengzhou 450046, China

**Keywords:** fruit quality, carotenoids, flavonoids, volatiles, tomato

## Abstract

Fruit quality is determined by multiple metabolites, which are mainly affected by plant hormones. In this study, two genes, ethylene-related gene *SlACO1* and gibberellin-related gene *SlGARP*, were overexpressed (OE) and knocked down through RNAi in tomato, and the profiles of carotenoids, flavonoids, volatiles, and hormones were detected in the leaves and fruit. The color index significantly increased in *SlACO1*-OE fruit but significantly decreased in the *SlACO1*-RNAi line. Similarly, total carotenoids, volatiles, salicylic acid, and ethylene significantly increased in the fruits of *SlACO1*-OE and *SlGARP*-OE, whereas ABA decreased significantly. Some compounds, such as lycopene, 3-hexenal, and *d*-limonene, significantly increased in the fruit of *SlACO1*-OE but decreased in the *SlACO1*-RNAi line, indicating that *SlACO1* might play an important role in the accumulation of these compounds. The functional characterization of SlACO1 and SlGARP would facilitate the improvement in tomato fruit quality.

## 1. Introduction

Quality-related compounds in horticultural crop plants consist of primary and secondary metabolites that contribute to nutritional value, appearance, color, and aroma. Many secondary metabolites within these compounds exhibit biological activities, including anti-cancer [1], free radical scavenging [2], anti-inflammatory [3], and anti-allergic [4] activities, as well as cardiovascular and cerebrovascular disease prevention [5]. These properties have attracted significant attention toward exploring the underlying mechanisms of fruit quality formation. Tomato, especially Micro-Tom, is an annual plant with a comparatively short growth cycle, efficient transformation capability [6], and a richness of terpenoids and flavonoids in the pericarp and pulp of its fleshy fruits. It has long been considered an excellent research model for studying primary and secondary metabolic pathways and their regulatory mechanisms [7]. Additionally, high-throughput mutagenesis and gene characterization are among the other notable characteristics of this plant [8,9].

In tomato, the functions of gibberellins (GAs) have primarily been reported in fruit set, parthenocarpy, plant height, and lateral branch development. However, few studies have focused on the role of GAs in fruit ripening. Changes in the biosynthesis of GAs also influence carotenoid metabolism, thereby affecting fruit color changes. The overexpression of PSY in tomato fruits led to excessive carotenoid accumulation in young fruits and dwarfism, and inhibited chlorophyll and GA_3_ biosynthesis [10]. By contrast, exogenous GA_3_ treatment downregulated the expression of *SlGA20ox2*, *SlGA20ox3*, and *SlGA30ox2*, as well as the cell division-related genes *SlCDKB1* and *SlCDKB2*, while upregulating that of gibberellin-inactivating genes *SlGA20ox1*, *SlGA20ox4*, and *SlGA20ox5*, along with cell enlargement genes *SlEXP2* and *SlEXP12* [11].

However, from the application of exogenous hormones to the regulation of fruit quality formation, many processes remain unclear, such as the efficiency of exogenous hormone dosage, absorption, transport, and degradation. Additionally, hormone response, signal transduction, regulation of target gene expression, and feedback effects on metabolites or genes require further explanation [12,13]. The ACO protein contributes to ethylene content in plants [14,15,16]. Ethylene-responsive factors play an important role in regulating carotenoid and fruit ripping metabolism [7,17]. GA biosynthesis and related genes are involved in the metabolism of carotenoids, volatiles, and chlorophyll contents [18,19]. ACO and GARP are important signaling genes in response to ethylene and GA, respectively, but their roles in the metabolism of tomato secondary metabolites remain unclear. In this study, by directly silencing or overexpressing the two hormone-responsive genes of ACO (*SlACO1*) and GA (*SlGARP*), we detected changes in metabolite profiles during fruit development and ripening. This approach provides direct evidence of stable hormonal regulation, independent of variables such as dosage, absorption, transport, and degradation efficiency, thereby presenting a consistent and direct hormone-mediated effect on fruit-quality-related metabolisms.

Since the completion of genome sequencing for many crops, functional genomics has become a new hotspot in recent years. Virus-Induced Gene Silencing (VIGS) is a transient gene-silencing technology that offers advantages such as easy induction of gene silencing in meristematic tissues, strong systemic spread, and mild symptom development [20]. However, overexpression phenotypes are less affected by functional redundancy and thus serve as a complementary approach to gene silencing for verifying gene function [21]. Therefore, the combined use of both overexpression and silencing constructs enables an effective in-depth screening and validation of gene functions.

In this study, Micro-Tom tomato was applied as a research material by using both VIGS and gene overexpression technologies, and metabolomics were employed to verify the roles of *SlACO1* and *SlGARP* in regulating terpenoids, carotenoids, flavonoids, and other quality-related secondary metabolites. Results from the present study may shed light on constructing the metabolic network of fruit quality regulated by hormone response genes.

## 2. Results

### 2.1. Isolation and Expression Analysis of SlACO1 and SlGARP in Tomato

The cDNA of *SlACO1* and *SlGARP* was cloned from tomato fruit, containing open reading frames of 948 and 315 bp, respectively. Based on protein phylogenetic tree analyses, *SlACO1* and *SlGARP* were identified as belonging to the 1-aminocyclopropane-1-carboxylate oxidase and gibberellin-regulated protein families, respectively (Figure S1).

**Figure 1:**
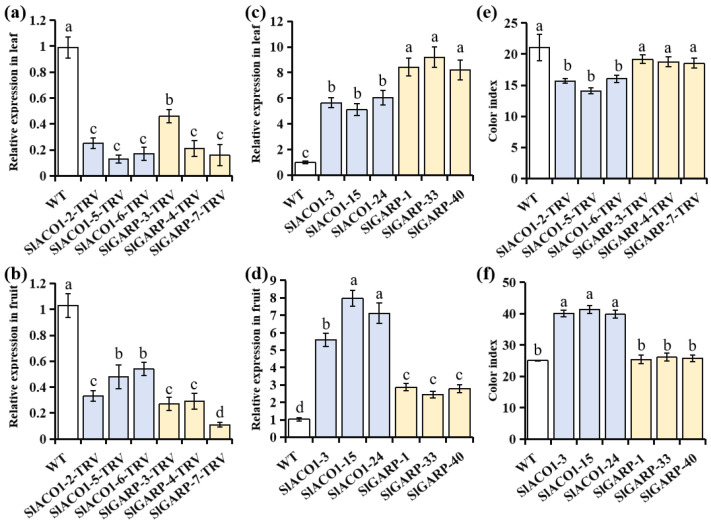
The relative expression levels of *SlACO1* and *SlGARP* in the tomato leaf (**a**) and fruit (**b**) RNAi line, respectively, and the tomato leaf (**c**) and fruit (**d**) overexpression line, respectively. The color index of fruit in the *SlACO1* and *SlGARP* RNAi (**e**) and overexpression lines (**f**), respectively. For bar charts, bars with different letters indicate significant differences analyzed by one-way ANOVA (with Tukey’s test; *p* < 0.05).

To verify the roles of *SlACO1* and *SlGARP* in tomato secondary metabolism, overexpression (OE) and RNAi vectors for both genes were transformed into Micro-Tom tomato. Transgenic lines were identified by PCR and RT-qPCR using gene-specific primers for *SlACO1* and *SlGARP* (Table S1), and these primers were used to detect specific bands in OE T_1_ lines (Figure S2). The RT-qPCR results showed that the expression levels in RNAi lines for both genes were significantly lower than in the WT (Figure 1a,b). By contrast, the expression levels of *SlACO1* and *SlGARP* in leaves were approximately 5- and 8-fold higher than in WT lines, respectively (Figure 1c), while in fruits, they were about 7- and 3-fold higher, respectively (Figure 1d).

### 2.2. Carotenoid Contents in SlACO1 and SlGARP Overexpression and RNAi Tomato

Carotenoids, which are natural pigments in fruits and vegetables, play a major role in determining fruit color. In our study, the CI value was significantly increased in the fruit of the *SlACO1*-OE line, but decreased in the RNAi line; however, no significant changes were observed in the fruits of *SlGARP*-OE and RNAi lines (Figure 1e,f).

In tomato leaves, a total of 12 carotenoids were detected (Table S5), with lutein and β-carotene being the most abundant components. Total carotenoid contents were significantly lower in the *SlACO1*-RNAi and *SlGARP*-RNAi lines compared to the WT, whereas *SlACO1*-OE and *SlGARP*-OE showed a significant increase relative to the WT. For each carotenoid content, compared with WT, violaxanthin and neoxanthin contents were significantly decreased in the *SlACO1*-RNAi and *SlGARP*-RNAi lines; β-carotene, lutein, and phytoene were significantly decreased in the *SlACO1*-RNAi lines, while for other compounds, there were no significant differences between the RNAi line and WT. By contrast, neoxanthin and lutein contents were significantly increased in the *SlACO1*-OE and *SlGARP*-OE lines (Table 1).

**Figure 2:**
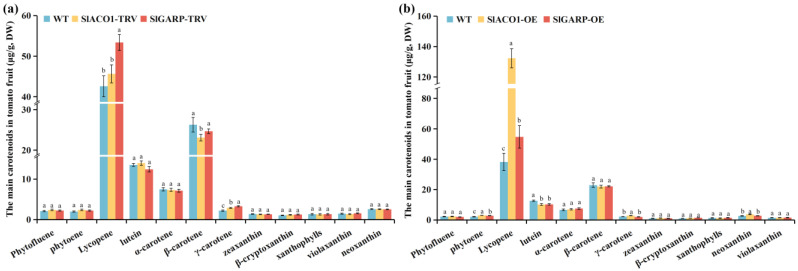
The contents of main carotenoids in (**a**) *SlACO1*-TRV and *SlGARP*-TRV tomato fruit and (**b**) *SlACO1* and *SlGARP* overexpression tomato fruit (μg/g DW). Different letters indicate significant differences analyzed by one-way ANOVA (with Tukey’s test; *p* < 0.05).

In tomato fruit, 14 carotenoids were determined (Table S6), among which lycopene and β-carotene were the most abundant compounds. Compared with the WT, the contents of violaxanthin, β-carotene, and lycopene contents were significantly decreased in *SlACO1*-RNAi fruit, while those of lycopene, neoxanthin, zeaxanthin, γ-carotene, and phytoene were significantly increased in *SlACO1*-OE fruit. The profiles of lycopene and γ-carotene were significantly decreased in the *SlGARP*-RNAi fruit but significantly increased in *SlGARP*-OE fruit (Figure 2).

### 2.3. Flavonoids in SlACO1 and SlGARP Overexpression and RNAi Tomato

In tomato leaves, 32 compounds were identified using LC-MS in positive and negative ion modes, including 6 amino acids and 26 phenolic acids and flavonoids. α-tomatine was the most abundant compound (13.54 ± 2.91 μg/g, DW), followed by phenylalanine (7.01 ± 0.85 μg/g, DW), tryptophan (6.36 ± 2.88 μg/g, DW), and leucine (4.34 ± 0.44 μg/g, DW); none of these showed significant differences among the WT, OE, and RNAi lines.

In tomato fruit, 54 compounds were determined, including 11 amino acids and 43 phenolic acids and flavonoids. Compared with the WT, the contents of five and three compounds were significantly altered in *SlACO1*-RNAi and *SlGARP*-RNAi line fruit, respectively. Among these, p-diphenylethylene was significantly reduced in both *SlACO1*-RNAi and *SlGARP*-RNAi lines (Table S2). As shown in Table 2, compared with the WT, SlACO1-OE fruits exhibited significantly increased levels of L-phenylalanine, 3-caffeoylquinic acid, kaempferol-3-O-rutinoside, and naringin 7-O-glucoside, while levels of chlorogenic acid, resveratrol, eriodictyol 7-O-glucoside/-hexose, kaempferol, α-tomatine, genistein, and naringenin were significantly decreased. In SlGARP-OE fruit, the contents of 3-caffeoylquinic acid, chlorogenic acid, α-tomatine, and genistein were significantly increased, whereas those of L-phenylalanine and naringenin were significantly decreased. Overall, the metabolic alterations induced by overexpression of these two hormone-responsive genes were more pronounced than those observed in the corresponding RNAi lines.

### 2.4. Volatile Compounds in SlACO1 and SlGARP Overexpression and RNAi Tomato

Volatile compound profiles in RNAi and OE tomato fruits were analyzed by GC-MS. A total of 17 compounds were identified, among which aldehydes represented the most abundant volatile class in both RNAi and OE lines, followed by acids, alcohols, and terpenes (Table S7; Figure S3).

The results indicated that aldehydes and acids were the predominant volatile compounds in tomato fruit. Compared with the WT, aldehyde contents were significantly decreased in the *SlACO1*-RNAi line but increased in the *SlGARP*-RNAi line. Acid contents were significantly increased in both *SlACO1*-RNAi and *SlGARP*-RNAi lines, while monoterpene contents were significantly decreased in *SlACO1*-RNAi. Relative to the WT, 13, 8, 11, and 9 volatile compounds showed significant changes in *SlACO1*-RNAi, *SlGARP*-RNAi, *SlACO1*-OE, and *SlGRAP*-OE fruits, respectively. In SlACO1-RNAi fruit, five compounds were significantly increased and eight were significantly decreased. In SlGARP-OE fruit, six were significantly increased and two were decreased. In SlACO1-OE fruit, six were increased and five were decreased, while in SlGARP-OE fruit, five were increased and four were decreased (Table 3).

For each compound, compared with the WT, 3-hecenal, *d*-limonene, and methylsalicylate were significantly decreased in *SlACO1*-RNAi fruit but increased in *SlACO*-OE fruit. By contrast, (E)-2-hexenal and octadecanoic acid were significantly increased in *SlACO1*-RNAi fruit but significantly decreased in *SlACO1*-OE fruit. Linolic acid and octadecanoic acid were significantly increased in *SlGARP*-RNAi fruit but decreased in *SlGRAP*-OE fruit (Table 3).

### 2.5. Hormone in SlACO1 and SlGARP Overexpression Tomato

Six hormones were detected in tomato fruit, among which the SA was the most abundant hormone, followed by free ABA as the second, and then by IAA, ZT, and so on, while JA was recorded with the lowest levels (Table 4). Compared with the WT, free ABA and SA contents were significantly decreased in the *SlACO1*-OE fruits, while JA and Eth were significantly increased; in *SlGARP*-OE fruits, free ABA, IAA, and JA contents were significantly decreased, while SA and Eth were significantly increased; however, ZT contents remained consistent with that of the control.

## 3. Discussion

### 3.1. Ethylene- and GA-Related Genes Affect the Accumulation in Metabolite Profiles

Plant hormones play versatile roles in plant growth regulation, and act as an important biochemical regulator with multiple biological activities, especially in the plant metabolites [22]. Previous studies have indicated that ethylene and GA signals play important role in the biosynthesis of carotenoids, volatiles, and flavonoids, ultimately affecting fruit quality [23]. SlACO1 and SlACO4 proteins contribute to ethylene content in tomato [14]. Ethylene regulates the expression of *PSY* and affects the accumulation of carotenoids in tomato and citrus fruits [24,25]; ethylene-responsive factors are involved in carotenoid metabolism regulation in plants [26,27,28]; similarly, GA biosynthesis reportedly affects the metabolism of carotenoids, volatiles, and chlorophyll contents [29]. In this study, the functions of ethylene-related gene *SlACO1* and GA-related gene *SlGARP* in the metabolism of volatiles, carotenoids, and flavonoids were identified using VIGS and overexpression technology in tomato leaves and fruits. Some compounds (β-carotene, kaempferol 3-O-rutin, valine, and 3-Hexenal) were significantly increased and decreased in overexpressed and RNAi tomato lines, respectively. However, some compounds (such as β-carotene and violaxanthin) showed a different accumulation pattern in fruit and leaf, likely due to the fact that target genes in leaf and fruit had a different expression pattern, or the expression level of the target genes might be affected by ethylene or GA, thereby acting on the main functional genes in the metabolic pathway of terpenoids and flavonoids, thus affecting their metabolic processes.

### 3.2. SlACO1 and SlGRAP Altered the Profiles of Carotenoids, Flavonoids, and Volatiles

Numerous molecular studies have shown that fruit ripening is the result of regulated functional gene expression [30], and lots of primary and secondary metabolic pathways are involved in the fruit ripening process, which play an important role in the overall sensory and nutritional quality of the fruit [31]. The total contents of volatile compounds were significantly increased in *SlGARP*-RNAi fruit; for volatile compounds, nine (3-hexenal, (E)-2-hexenal, α-pinene, (E)-2-heptenal, β-pinene, dodecane, n-hexadecanoic acid, linolic acid, and octadecanoic acid) of them were significantly changed in *SlGARP*-RNAi fruit. Similar to *SlGARP*-RNAi fruit, there were 13, 11, and 9 volatiles which were significantly changed in *SlACO1*-RNAi, *SlACO1*-OE, and *SlGRAP*-OE fruits, respectively (Table 3). Combining both the silenced and overexpressed fruits, there were five (3-hexenal, (E)-2-hexenal, *d*-limonene, methylsalicylate, and octadecanoic acid) and two (linolic acid and octadecanoic acid) compounds showing an opposite pattern in *SlACO1* and *SlGARP* fruit, respectively. Therefore, the hormone-related genes of *SlACO1* and *SlGARP* may play an important role in the metabolism of those five and two volatile compounds in tomato fruit, respectively.

Furthermore, some terpenoids, such as β-carotene, γ-carotene, and lycopene, and some monoterpenes showed great differences in RNAi and OE lines; for example, lycopene and γ-carotene were significantly increased in *SlACO1*-RNAi fruit, as well as some monoterpenes, acids, and flavonoids. To date, the regulation network of terpenoids and flavonoids is still in a preliminary stage, especially for the flavonoids. The regulation network of terpene biosynthesis is complex, both because of transcription factors and environmental factors, such as temperature and light. Light signals are involved in the regulation of PSY expression level, which involves total carotenoid content accumulation [32]. Amino and fatty acids were the major biosynthesis pathways for volatiles; PAL and LOX were the major rate-limiting enzymes for aldehyde biosynthesis [33]; and CHS, CHI, and F3H were the key genes for flavonoid biosynthesis [34]. In this study, the carotenoids, flavonoids, and volatiles were significantly changed in transgenic *SlACO1* and *SlGARP* (overexpression and RNAi) tomato fruits, indicating that *SlACO1* and *SlGARP* may be involved in the biosynthesis of these three pathways, but the target genes still need to be further identified using LUC, Y1H, and EMSA.

### 3.3. GA and Ethylene Signals Affect Metabolites in Fruits

Previous studies have indicated that the expression level of the *PSY* gene was enhanced in tomato fruits treated with ethephon, and as a result, the up- and downstream synthesis of lycopene was increased and inhibited, respectively [35]. A considerable number of reports show that ABA and ethylene exhibit different interaction patterns in different physiological processes. For example, CsbZIP44 directly binds to and activates the promoters of carotenoid (CsDXR, CsGGPPs, and CsBCH1) and ABA (CsNCED2) metabolism-related genes; furthermore, CsHB5-CsbZIP44 protein complex responds to ABA induction and increases carotenoid accumulation in citrus [36]. Two ethylene response factors, CsERF110 and CsERF53, are induced by ABA signals and significantly influence carotenoid metabolism in citrus [27]. The GA signal inhibits carotenoid accumulation by influencing the protein abundance of MiGAIP1 [19]. In addition, GA plays an important role in volatile accumulation in petunia [37] and Arabidopsis [38]. In this study, we found that some carotenoids and volatiles were significantly affected in *SlACO1* and *SlGARP* transgenic tomatoes. To sum up, the relationship between GA_3_ and ethylene is a complex regulatory network, which exhibits both crossing and overlapping.

As we know that fruit development is a complex biological process, it is difficult to study the effect of one hormone in isolation, so it is imperative to attempt studying the interaction of two or more hormones. In the near-future, using expression chip analysis, proteomics, and functional genomics tools, we will be able to analyze gene expressions in fruit development more comprehensively; in turn, the linkage between different metabolic pathways and signaling pathways could be explored. In addition, we can also learn about the spatiotemporal information of these gene expressions, including the blueprint for systems responding to environmental factors, hormones, and other growth regulators.

## 4. Materials and Methods

### 4.1. Materials

Micro-Tom tomato (*Lycopersicon esculentum*) seeds were purchased from Fengshuo Horticultural Co., Ltd. (Nanjing, China), and Trans5α (*E. coli*) and the pMD18-T vector were purchased from Beijing Dingguo Changsheng Biotech Co., Ltd. (Beijing, China). The strains pH2WG2D and GV3101 (*Agrobacterium tumefaciens*) were kept in our laboratory, while EHA105 was acquired from Biosgene Co., Ltd. (Wuhan, China).

MS powder medium and antibiotics, such as IAA and zeatin riboside, were purchased from Beijing Dingguo Changsheng Biotech Co., Ltd. (Beijing, China). Trizol buffer for total RNA extraction was purchased from Aidlab Biotechnologies Co., Ltd. (Beijing, China). The RNA reverse transcription kit, n-hexane, acetone, absolute alcohol, acetic acid, ethyl nonate, methyl tert-butyl ether (MTBE), acetonitrile, and methanol were purchased from Thermo Fisher Scientific Co., Ltd. (Waltham, MA, USA). Acetosyringone (AS), MES-2-(N-Morpholino) ethanesulfonic acid, magnesium chloride (MgCl_2_), lidocaine, BHT, NaCl, and KOH were purchased from Sigma-Aldrich (St. Louis, MO, USA).

### 4.2. RNA Extraction and RT-qPCR Analysis

RNA was extracted from tomato leaves and fruit using the RNA extraction kit (RNAiso, TAKARA, Kyoto, Japan) according to standard protocols. An aliquot of 1.5 μg of total RNA was subjected to cDNA synthesis, and the first-strand cDNA was synthesized using the Revert Aid First Strand cDNA Synthesis Kit (K1622, Thermo Fisher Scientific). The gene-specific primers used for RT-qPCR are listed in Table S1. RT-qPCR was performed using the ABI 7500 system with 384-well plates using the Hieff^TM^ qPCR SYBR Green Master Mix (No Rox, Yeasen Biotech Co., Ltd., Shanghai, China), and the program was performed according to the manufacturer’s protocol. *Actin* was used as the endogenous control. RT-qPCR data analysis was performed as described by [39].

### 4.3. Vector Construction and Tomato Transformation

The full CDS sequences of *SlACO1* and *SlGARP* were cloned from the tomato fruit, of which the primers are listed in Table S1, and then the fragments were cloned into the pTOPO-blunt vector, respectively. The CDS sequences of *SlACO1* and *SlGARP* were constructed into the pH2WG2D vector according to the Gateway method, and then, the destination vector was transferred into EHA105 *Agrobacterium tumefaciens* according to previous protocols [40]. The specific coding region sequences of *SlACO1* and *SlGARP* were predicted using SGN VIGS Tools (https://vigs.solgenomics.net/, accessed on 3 January 2026), and then the restriction sites were analyzed using Webcutter 2.0 (http://heimanlab.com/cut2.html, accessed on 3 January 2026). Those specific sequences were also cloned and then constructed into vector TRV as per established protocols [41]. The final vectors were transferred into GV3101 *Agrobacterium tumefaciens* using a heat shock method. According to the method described in a previous study [42], a single bacterial colony containing the vector (*SlACO1*-RNAi and *SlGARP*-RNAi) was used for inoculation in liquid LB medium, and grown at 28 °C until the absorbance reached 1.2–2.0 at 600 nm. The tomato cotyledon and the fruit stem at 10 d DAF were used to inject the vector of *SlACO1*-RNAi and *SlGARP*-RNAi, respectively. The RNAi tomato lines were living at 19 °C under 30% relative humidity and 16/8 h light/dark conditions. The *Agrobacterium tumefaciens* containing the overexpressed vectors of *SlACO1* and *SlGARP* were used for transformation in tomato as per previous methods [43]. The TRV and overexpressed transgenic tomatoes were identified using PCR and confirmed by RT-qPCR using gene-specific primers (Table S1).

### 4.4. Carotenoid Extraction and HPLC Analysis

The carotenoids were extracted from a 0.1 g sample of tomato leaf, or 1.5 g of tomato fruit samples, and the method adopted was according to [44]. The extraction was detected using Waters 1525 high-performance liquid chromatography (HPLC, Waters Corp., Milford, MA, USA) combined with the 2996 photodiode array detector, and using the YMC column (4.6 mm × 150 mm × 5 μm, C30, Wilmington, NC, USA). For compound identification, five carotenoid standards were employed, and the carotenoid contents were quantified based on the standard curves according to our previous studies [44].

### 4.5. Flavonoid Extraction and LC-MS Analysis

Flavonoid extraction was performed according to the method described by [34] with minor modifications. The flavonoids were extracted from 0.1 g of leaf or fruit using 2 mL of 80% methanol (80/20, *v*/*v*, containing 0.1 mg/L of lidocaine as an internal standard), and then, the extraction was carried out using an ultrasonic bath (Thermo Fisher Scientific) at 40 °C for 30 min. The mixture was centrifuged for 10 min, and the supernatant was filtered using the 0.22 µm membrane prior to LC-MS analysis. The flavonoids were detected using HPLC-DAD-ESI-QqTOF-MS/MS (6520B, Agilent, Santa Clara, CA, USA) combined with a ZORBAX C18 MS column (2.1 × 100 mm × 1.8 μm). Flavonoid identification and quantitative analysis was performed according to [34].

### 4.6. Volatile Extraction and Identification

Volatile extraction was performed according to [45] with minor modifications. A fresh tomato sample of 1 g was used for each sample, and methyl nonoate served as the internal standards [45]. The volatiles were measured using GC-MS (TRACE GC Ultra GC) coupled with the DSQ II mass spectrometer (Thermo Fisher Scientific) in splitless mode. Other parameters were maintained according to [46]. For compound identification, the raw data were identified based on the NIST mass spectral library (NIST 2015) using Xcalibur software (version 4.3, Thermo Fisher Scientific) [45]. The concentrations of individual volatile compounds were quantified based on the internal standard.

### 4.7. Extraction and Determination of Endogenous Hormone

Quantitative detection of IAA, ZT, salicylic acid (SA), jasmonic acid (JA), abscisic acid (ABA), and ethylene (Eth) in tomato fruit was measured by UPLC-ESI-MS/MS in Shanghai Luming Biotechnology Co., Ltd. (Shanghai, China). Amounts of 10 mg of lyophilized samples were extracted using 80% methanol, and then, the mixture was immersed in an ultrasonic bath model FS60 (Fisher Scientific, Pittsburgh, PA, USA) for 30 min at 4 °C. The extract was centrifuged at 13,000 RPM for 5 min, and the extract was filtered using a 0.22 μm membrane prior to analysis. The parameters for UPLC-ESI-MS/MS were optimized and set at a cone voltage of 30 V, collision voltage of 8 V, source offset voltage of ±3 kV, and desolvation temperature of 500 °C. By contrast, other parameters were set according to [47]. A Waters ACQUITY UPLC BEH C18 (100 mm × 2.1 mm × 1.7 μm) column was employed for separation, the injection volume was 5 μL, and the mass spectrometry parameters are listed in Table S3. Water (0.1% methanoic acid) and acetonitrile (0.1% methanoic acid) were used as the solvent system, and the gradient program is shown in Table S4.

### 4.8. Color Index

The color difference (ΔE) of tomato fruits in the mature stage was measured using a CHROMA METER CR-400 (Monolta, Tokyo, Japan), six fruits were measured for each biological repetition, and three points for each fruit were measured, finally obtaining the *L*, *a*, and *b* values. The color index (CI) was calculated using the following formula:

$$\mathrm{Color}\;\mathrm{index}\;=\frac{1000\times a}{L\times b}\;(a>0\;\&\;b>0)$$

*L*: lightness; *a*: greenness to redness; *b*: blueness to yellowness.

### 4.9. Data Analysis

Volatiles, flavonoids, carotenoids, and hormones were measured at three biological replicates, the contents of which were calculated using Microsoft Excel 2010 (USA) based on standard curves or an internal standard. The mean and standard deviation (SD) values were calculated using Microsoft Excel 2010 (USA) and are listed in the tables and figures. SAS 8.0 software (SAS Institute Inc., Cary, NC, USA) was employed for the analysis of variance (ANOVA) (*p* < 0.05).

## 5. Conclusions

Fruit quality directly affects its economic value and consumer selection, while secondary metabolites, such as volatiles, carotenoids, and flavonoids, contribute to fruit quality. Two hormone-related genes, *SlACO1* and *SlGARP*, were overexpressed and knocked down in tomato, and the results revealed that carotenoids, volatiles, SA, and ethylene were significantly increased, while ABA was significantly decreased, in the fruit of *SlACO1*-OE and *SlGARP*-OE lines. Similarly, lots of volatiles, carotenoids, and flavonoids were significantly changed in the overexpressed lines, indicating that *SlACO1* and *SlGARP* genes play important roles in the accumulation of carotenoid, volatile and flavonoid compounds, thereby affecting tomato fruit quality.

## Figures and Tables

**Table 1:** The contents of carotenoids in tomato leaves (μg/g, DW *p* < 0.05).

Construct	Violaxanthin	Neoxanthin	Lutein	β-Carotene	Phytoene	Total
WT	0.67 ± 0.06 a	1.64 ± 0.02 a	5.48 ± 0.09 a	3.72 ± 0.32 a	0.37 ± 0.08 a	11.88 ± 1.20 a
SlACO1-TRV	0.10 ± 0.01 b	1.10 ± 0.10 b	4.82 ± 0.26 a	3.30 ± 0.27 a	0.34 ± 0.08 a	9.66 ± 0.72 b
SlGARP-TRV	0.07 ± 0.01 b	0.28 ± 0.05 c	2.23 ± 0.17 b	1.49 ± 0.19 b	0.23 ± 0.01 b	4.30 ± 0.46 c
WT	1.00 ± 0.08 a	0.43 ± 0.02 c	4.10 ± 0.06 b	2.00 ± 0.20 a	0.70 ± 0.07 a	8.20 ± 0.97 b
SLACO1-OE	0.95 ± 0.01 a	0.60 ± 0.02 b	9.20 ± 0.14 a	2.10 ± 0.03 a	0.77 ± 0.01 a	13.0 ± 0.21 a
SlGARP-OE	1.00 ± 0.10 a	0.89 ± 0.08 a	9.60 ± 0.80 a	2.00 ± 0.15 a	0.74 ± 0.04 a	14.0 ± 0.45 a

Different letters indicate significant differences analyzed by one-way ANOVA (with Tukey’s test; *p* < 0.05).

**Table 2:** The compounds were measured in the *SlACO1* and *SlGARP* overexpression fruit (μg/g, DW).

Compounds	*m*/*z*	Formula	WT	SlACO1-OE	SlGARP-OE
L-Phenylalanine	166.1	C_9_H_11_NO_2_	27.11 ± 1.70 b	41.23 ± 2.90 a	2.90 ± 1.20 c
3-Caffeoylquinic acid	355.1	C_16_H_18_O_9_	0.20 ± 0.01 c	2.30 ± 0.26 b	4.90 ± 0.30 a
Chlorogenic acid	354.3	C_16_H_18_O_9_	0.55 ± 0.09 b	ND c	0.86 ± 0.07 a
Resveratrol (3,5,4′-tri-hydroxystilbene)	228.2	C_14_H_12_O_3_	1.00 ± 0.15 a	0.11 ± 0.02 b	ND c
Rutin	611.1	C_27_H_30_O_16_	6.50 ± 0.58 a	7.30 ± 0.19 a	ND b
Eriodictyol 7-O-glucoside/-hexose	451.1	C_21_H_22_O_11_	0.57 ± 0.07 a	ND b	0.70 ± 0.20 a
Naringenin 7-O-glucoside	435.1	C_21_H_22_O_10_	0.10 ± 0.01 b	0.57 ± 0.02 a	0.15 ± 0.33 b
Kaempferol-3-O-rutinoside	594.5	C_27_H_20_O_12_	2.60 ± 0.14 b	3.20 ± 0.12 a	3.50 ± 0.30 a
Kaempferol	286.2	C_15_H_10_O_6_	7.10 ± 0.53 a	0.37 ± 0.08 b	ND c
α-Tomatine	1034.5	C_50_H_83_NO_21_	2.30 ± 0.11 b	1.88 ± 0.12 c	4.70 ± 0.40 a
Genistein	270.2	C_15_H_10_O_5_	0.62 ± 0.05 b	ND c	18.6 ± 3.50 a
Naringenin	272.2	C_15_H_12_O_5_	8.90 ± 1.50 a	1.82 ± 0.17 c	4.40 ± 0.46 b

Different letters indicate significant differences analyzed by one-way ANOVA (with Tukey’s test; *p* < 0.05).ND: Not detected.

**Table 3:** The contents of volatiles in tomato fruits (μg/g, FW).

Compound	WT-TRV	SlACO1-TRV	SlGARP-TRV	WT-OE	SlACO1-OE	SlGARP-OE
3-Hexenal	6.20 ± 0.59 b	1.81 ± 0.13 c	10.80 ± 0.28 a	14.0 ± 0.30 c	29.0 ± 1.10 a	19.60 ± 3.20 b
(E)-2-Hexenal	2.37 ± 0.21 b	2.73 ± 0.01 a	3.14 ± 0.50 a	4.34 ± 0.23 b	1.70 ± 0.02 c	11.10 ± 1.60 a
α-Pinene	0.12 ± 0.03 a	0.03 ± 0.03 b	0.07 ± 0.05 b	0.92 ± 0.10 a	0.45 ± 0.02 b	ND c
(E)-2-Heptenal	Trace b	ND b	0.14 ± 0.05 a	Trace	Trace	Trace
β-Pinene	0.16 ± 0.06 a	ND b	ND b	ND	ND	ND
β-Myrcene	0.45 ± 0.12 a	0.08 ± 0.02 b	0.36 ± 0.08 a	ND b	ND b	0.49 ± 0.04 a
d-Limonene	0.40 ± 0.44 a	0.08 ± 0.05 b	0.39 ± 0.53 a	Trace b	1.30 ± 0.05 a	Trace b
β-cis-Ocimene	0.04 ± 0.02 a	ND b	0.03 ± 0.03 a	Trace b	ND b	0.36 ± 0.04 a
Undecane	0.08 ± 0.00 a	ND b	0.07 ± 0.00 a	0.58 ± 0.07 a	0.18 ± 0.09 c	0.43 ± 0.01 b
Dodecane	ND c	0.14 ± 0.00 b	0.40 ± 0.01 a	0.31 ± 0.01 a	0.32 ± 0.02 a	0.30 ± 0.01 a
Methylsalicylate	0.34 ± 0.01 a	Trace b	0.26 ± 0.02 a	0.12 ± 0.01 b	0.27 ± 0.02 a	0.17 ± 0.04 b
(E,E)-2,4-Decadienal	Trace	ND	Trace	0.25 ± 0.01 b	1.39 ± 0.09 a	Trace c
Nerolidol	ND	ND	Trace	1.70 ± 0.02 a	0.32 ± 0.03 b	0.15 ± 0.01 c
n-Hexadecanoic acid	1.25 ± 0.66 b	2.64 ± 1.14 a	2.12 ± 0.67 a	0.06 ± 0.01 c	1.04 ± 0.21 b	1.90 ± 1.10 a
Linolic acid	2.53 ± 0.38 c	5.12 ± 0.60 b	7.82 ± 1.96 a	0.53 ± 0.08 b	10.0 ± 1.90 a	Trace c
Octadecanoic acid	0.23 ± 0.15 b	1.84 ± 0.34 a	1.63 ± 0.22 a	3.20 ± 0.03 a	0.58 ± 0.07 b	0.17 ± 0.06 c
Total	15.24 ± 2.90 b	15.47 ± 3.2 b	28.32 ± 4.76 a	26.12 ± 2.30 c	48.36 ± 3.50 a	35.50 ± 5.30 b

Note: -TRV: VIGS fruits. -OE: Gene overexpression in fruits. ND: Not detected; Different letters indicate significant differences analyzed by one-way ANOVA (with Tukey’s test; *p* < 0.05).

**Table 4:** Phytohormone contents in tomato fruit of *SlACO1* and *SlGARP* lines (ng/g, DW).

Construct	IAA	ZT	ABA	SA	JA	Eth	GA3
WT	8.84 ± 0.94 a	1.39 ± 0.06 a	97.36 ± 1.01 a	131.73 ± 5.77 c	0.58 ± 0.03 b	0.87 ± 0.38 c	3.54 ± 0.32 b
SlACO1-OE	8.11 ± 1.14 a	1.61 ± 0.12 a	69.60 ± 3.36 b	110.38 ± 12.68 b	0.97 ± 0.15 a	1.63 ± 0.27 b	3.88 ± 0.34 b
SlGARP-OE	5.19 ± 0.75 b	1.16 ± 0.11 a	64.62 ± 2.24 b	297.73 ± 106.13 a	0.38 ± 0.04 c	2.88 ± 0.34 a	4.51 ± 0.28 a

Different letters indicate significant differences analyzed by one-way ANOVA (with Tukey’s test; *p* < 0.05).

## Data Availability

The original contributions presented in this study are included in the article/Supplementary Materials. Further inquiries can be directed to the corresponding author(s).

## Supplementary Materials

The supporting information can be downloaded at: https://www.mdpi.com/article/10.3390/ijms27021078/s1.
