# Supplementary material for: SlACO1 and SlGARP Regulate Hormone-Mediated Metabolic Profiles in Tomato Fruit
Source: Int J Mol Sci. 2026 Jan 21;27(2):1078. doi: 10.3390/ijms27021078 (PMC12842259; doi:10.3390/ijms27021078)
Supplement: Supplementary file 1 [file ijms-27-01078-s001.zip › Supplementary Figures.pdf]

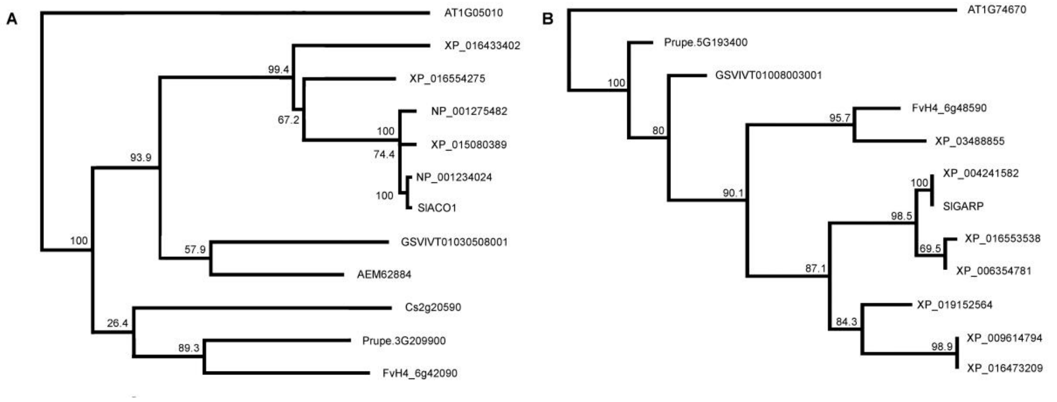

Figure S1. Phylogenetic tree analyses of SlACO1 (A) and SlGARP (B).

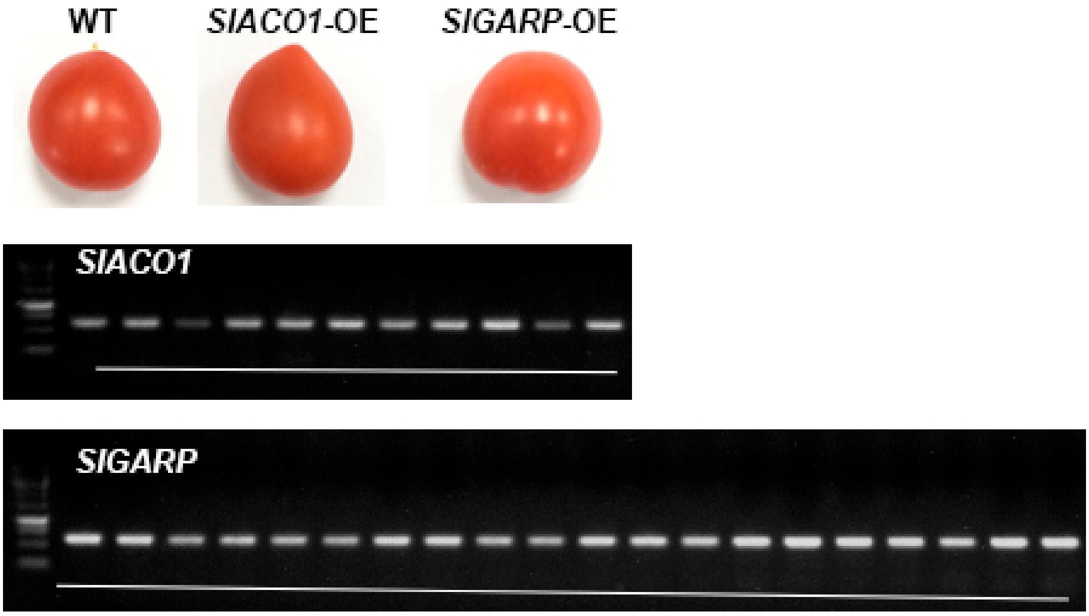

Figure S2. Photographs of fruits and PCR result for SlACO1 and SlGARP transgenic lines.

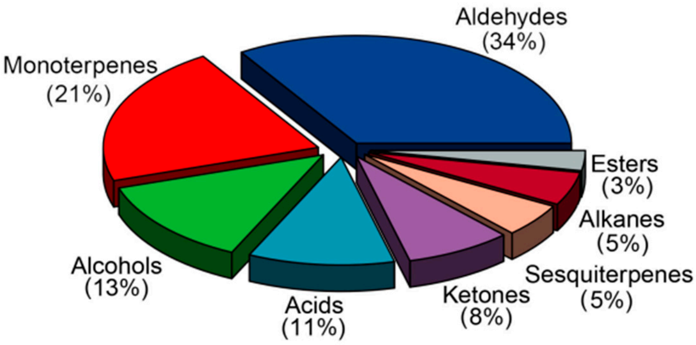

Figure S3. Volatile constitutions in the WT tomato fruits.
